# Genome Sequence of *Stenotrophomonas maltophilia* Strain SmAs1, Isolated From the Asian Malaria Mosquito *Anopheles stephensi*

**DOI:** 10.1128/genomeA.00086-16

**Published:** 2016-03-10

**Authors:** Grant L. Hughes, Juan Antonio Raygoza Garay, Vikas Koundal, Jason L. Rasgon, Michael M. Mwangi

**Affiliations:** a Department of Pathology and Institute for Human Infections and Immunity, University of Texas Medical Branch, Galveston, Texas, USA; b Department of Biochemistry and Molecular Biology, Center for Infectious Disease Dynamics, Pennsylvania State University, University Park, Pennsylvania, USA; c Huck Institutes of the Life Sciences, Pennsylvania State University, University Park, Pennsylvania, USA; d Department of Entomology, Center for Infectious Disease Dynamics, Pennsylvania State University, University Park, Pennsylvania, USA

## Abstract

An isolate of *Stenotrophomonas maltophilia* was cultured from the Asian malaria vector *Anopheles stephensi*. Here, we present the annotated draft genome sequence of this *S. maltophilia* strain. This genomic resource will facilitate further characterization of bacteria associated with mosquitoes.

## GENOME ANNOUNCEMENT

Mosquitoes are known to harbor a diverse microbiome (1–4), which can influence many host phenotypes (5). Importantly, from a public health perspective, bacterial microbes are known to influence the ability of *Anopheles* mosquitoes to transmit both viral and apicomplexan pathogens (6–8), and microbial control approaches to controlling arthropod-borne disease are gaining considerable attention. While our appreciation of the influence of the microbiome on mosquitoes is expanding, there are limited genomic resources for bacteria that associate with the Asian malaria mosquito. Here, we report the draft genome sequence of a *Stenotrophomonas maltophilia* isolate, cultured from laboratory-reared *Anopheles stephensi* mosquitoes.

A homogenate of surface-sterilized *A. stephensi* (Liston strain) was used to inoculate LB agar plates, and a single colony was isolated and confirmed by 16S rRNA gene sequencing to be *S. maltophilia*. Genomic DNA was extracted using a Qiagen Blood and Tissue kit following the recommendation for bacteria. The sequencing was done in a 500-cycle run on an Illumina MiSeq at the Pennsylvania State University Genomics Core Facility. The DNA library was prepared using a Nextera XT DNA library preparation kit and had an insert size of 400 bp. The 250-bp paired-end reads were initially assembled using MIRA version 4.0, and the assembly was refined using DNAStar SeqMan Pro version 12.0. This resulted in a total of 32 contigs with a combined length of 4.0 Mbp, an *N*_50_ statistic of 166,372 bp, a median read coverage of 66×, and an average G+C content of 66%. The annotation was done using the RAST pipeline (9–11), followed by manual curation, yielding 3,625 protein-coding genes and 67 RNA genes.

One species of *Stenotrophomonas*, namely, *S. maltophilia*, is an emerging global multidrug-resistant opportunistic human pathogen (12). The RAST pipeline identified genes with homology to resistance proteins: multidrug/efflux pumps, 49; heavy metal-resistance proteins, 48; beta-lactamases, 10; acriflavin-resistance proteins, 3; macrolide efflux pumps, 3; aminoglycoside acetyltransferases, 2; aminoglycoside phosphotransferases, 1; fosmidomycin-resistance proteins, 1; and fusaric acid-resistance proteins, 1.

*Stenotrophomonas* spp. appear to associate with multiple insect species (13–16). The bacterial genome sequences in this and other papers (17, 18) will provide further resources to examine bacterial interactions within insects.

### Nucleotide sequence accession number.

This whole-genome shotgun project has been deposited at GenBank under the accession number LFKU00000000.
